# Regional impairment of left ventricular longitudinal strain in aortic regurgitation

**DOI:** 10.1186/s44348-024-00028-z

**Published:** 2024-09-05

**Authors:** Joana Ferreira, Liliana Marta, João Presume, Pedro Freitas, Sara Guerreiro, João Abecasis, Carla Reis, Regina Ribeiras, Miguel Mendes, Maria João Andrade

**Affiliations:** 1 Department of Cardiology, Hospital de São Bernardo, Centro Hospitalar de Setúbal, Setúbal, Portugal; 2 Department of Cardiology, Hospital de Santa Cruz, Centro Hospitalar Lisboa Ocidental, Carnaxide, Portugal

**Keywords:** Aortic valve insufficiency, Myocardial contraction, Global longitudinal strain

## Abstract

**Background:**

Aortic regurgitation (AR) has an important impact on myocardial mechanics and recent studies have proved the value of global longitudinal strain (GLS) in the assessment of its severity and prognosis. Our purpose was to assess if the direct impact of the regurgitant jet on the myocardial wall could affect regional longitudinal strain.

**Methods:**

Eighty patients with chronic moderate/severe AR were retrospectively studied. Patients were considered to have a jet-related longitudinal strain reduction when the myocardial segments directly impacted by the jet had their longitudinal strain reduced by at least 30% compared to nonaffected segments. AR severity, left ventricular (LV) size and function were compared according to the presence/absence of this regional pattern. For those who underwent surgery, postoperative regional and global LV function was also analyzed.

**Results:**

A pattern of regional longitudinal strain impairment was identified in 43% of patients, with a regional reduction (in median) of 10 percentage points in absolute strain values in the segments impacted by the jet, compared to nonaffected segments. In the subgroup who underwent surgery, this pattern became attenuated after surgery. Patients with regional longitudinal strain impairment were less likely to improve GLS after surgery (10% vs. 38% improved GLS by at least 2.5%, *P* = 0.049).

**Conclusions:**

To our knowledge, this study identifies for the first time, a link between the location of the impact of the regurgitant jet in AR and regional longitudinal strain impairment. The presence of this regional pattern might be associated with worse postoperative LV recovery.

## Background

Aortic regurgitation (AR) is a common but often asymptomatic valvular disease which has a direct impact over the structure and function of the left ventricular (LV) myocardium. It is associated with both pressure and more importantly, volume overload which, when severe, results in marked ventricular remodeling with eccentric hypertrophy, ultimately leading to the development of interstitial and regional replacement fibrosis [1, 2].

Current guidelines recommend intervention when AR becomes symptomatic or is associated with reduced LV ejection fraction (LVEF) or LV dilatation [3]. However, myocardial deformation analysis using speckle tracking has shown that impairment of longitudinal systolic function can occur while the patient is still asymptomatic, with preserved LVEF. Recent studies have proved the value of global longitudinal strain (GLS) as an additional parameter in the assessment of disease severity and prognosis. Namely, a lower GLS has been found to be correlated with disease progression and mortality in patients treated conservatively and also to be associated with worse outcome after surgical correction of AR [4–6]. However, a direct and localized influence of the aortic regurgitant jet on regional deformation has only been described once, in a case report [7].

Therefore, the main purpose of this study was to assess if there is a regional pattern of impairment of LS in AR related to the impact of the regurgitant jet and whether it is reversible with surgical correction of the regurgitation. Additionally, we aimed to evaluate the association between this regional pattern and the recovery of LV systolic function after surgery.

## Methods

### Ethics statement

The study was conducted in compliance with the ethical principles outlined in the Declaration of Helsinki. Considering its observational and retrospective nature, Institutional Review Board approval was not necessary for this study at our institution and the need for informed consent was waived.

### Population and study design

This retrospective study included patients with chronic moderate or severe AR referred for echocardiographic evaluation in a tertiary hospital between May 2009 and November 2021. Exclusion criteria were the presence of concomitant moderate or severe valve disease (including aortic stenosis), cardiomyopathy, left bundle branch block, history of coronary artery disease and a poor acoustic window. Demographical and clinical data were extracted from the patients’ electronic medical record and echocardiographic data from the echocardiography laboratory image archive. All data were anonymized before analysis.

Patients were divided into two groups according to whether a pattern of jet-related LS reduction was identified in the echocardiographic assessment. For those who underwent surgical correction of AR, data from the postoperative echocardiogram closest to 6 months postsurgery was also collected.

### Echocardiographic analysis

Two-dimensional (2D) transthoracic echocardiograms (TTE) were performed using a commercially available ultrasound machine (Vivid 7 and Vivid E95, GE HealthCare), stored and analyzed using the software package EchoPac ver. 204 (GE Ultrasound). Standard 2D, Doppler, and tissue Doppler assessments were performed.

AR severity was estimated using the multiparametric approach recommended in the European Association of Cardiovascular Imaging guidelines [8], including effective regurgitant orifice area, regurgitant volume, and pressure half-time. LV linear dimensions were measured in parasternal long-axis view. LV volumes and EF were assessed using the biplane Simpson method from the apical two- and four-chamber views.

#### Segmental and global LV LS

LV LS analysis was performed offline at the workstation by a blinded cardiologist, using the apical four-, three-, and two-chamber views acquired by a sonographer or cardiologist. The region of interest was defined semiautomatically and manually adjusted when necessary. When myocardial tracking was deficient in more than one myocardial segment, the patient was excluded. GLS was calculated automatically from the average of the LS of all myocardial segments.

#### Baseline TTE assessment

**Fig. 1:**
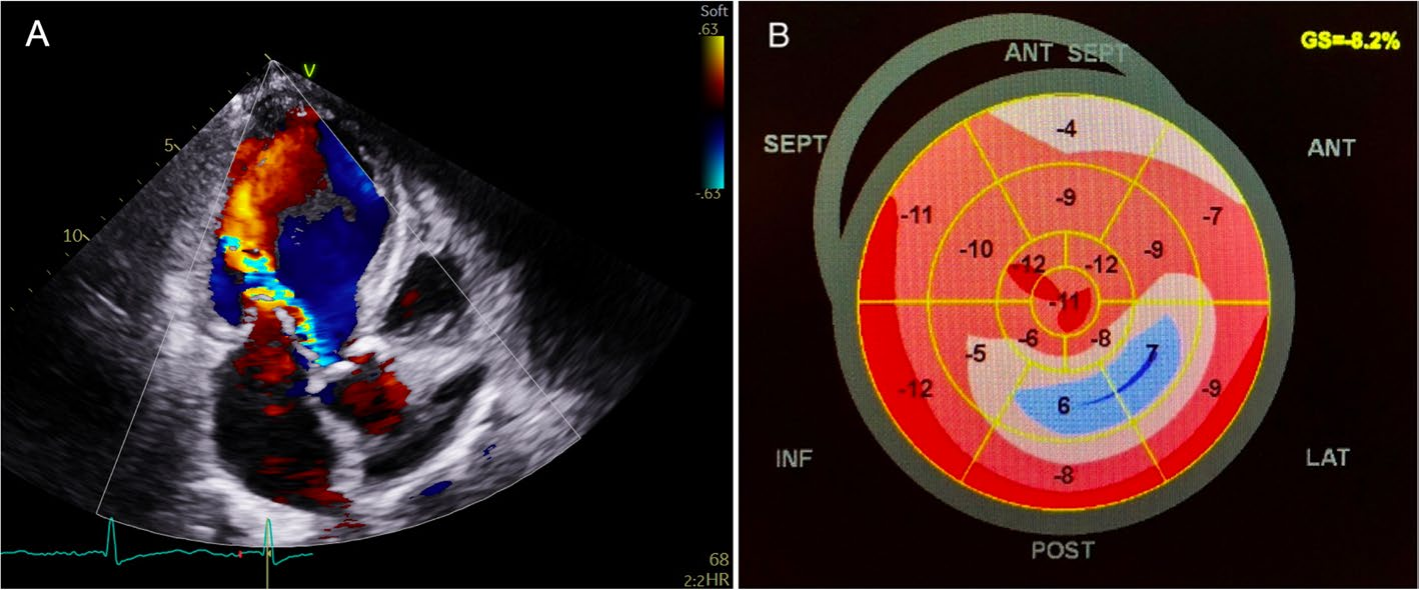
Example of a case of aortic regurgitation associated with jet-related strain reduction. (**A**) Apical three-chamber view showing an aortic regurgitant jet directed towards the mid inferolateral segment. (**B**) Longitudinal strain bull’s eye of the same patient showing worse longitudinal strain values in the mid inferolateral and anterolateral segments (impacted by the jet)

In addition to the above-mentioned measurements, for each patient, we assessed the direction of the aortic regurgitant jet and defined it according to the myocardial segments which were impacted by the turbulent portion of the jet (as described in the example displayed in Fig. 1). The segmental LS values were then evaluated. Patients were considered to have a jet-related LS reduction when the myocardial segments directly impacted by the jet had a relative reduction in LS of at least 30% compared with segments not affected by the jet. Thirty percent was chosen as cutoff considering the 10% test–retest variability for segmental LS in GE devices [9].

#### Postoperative TTE assessment

The difference in LV volumes, EF, and GLS between preoperative and postoperative assessments was calculated and compared between the two groups. Similarly to previous studies [10, 11], an absolute increase in LVEF > 5% compared with the preoperative value was necessary for an improvement in LVEF to be considered relevant. Patients were considered to have improved GLS if the absolute LS value increased (i.e., became more negative) by at least 2.5% since the test–retest variability in GE devices is estimated at < 1.5% absolute error in GLS values [12].

### Statistical analysis

Categorical variables were expressed as frequencies and percentages and were compared using the chi-square test or Fisher test, when appropriate. Continuous variables were assessed for normality with the Kolmogorov–Smirnov test. Variables were presented as mean and standard deviation in case of a normal distribution, and as median and interquartile range in the absence of normality. They were compared with Student t-test or Mann–Whitney test, respectively. Statistical analysis was performed using IBM SPSS ver. 23.0 (IBM Corp) and a two‐tailed *P*‐value of < 0.05 was considered statistically significant.

## Results

### Study population

Of the 203 patients screened, 123 fulfilled exclusion criteria, which resulted in a final sample of 80 patients, predominantly male (83%), with a mean age of 56 ± 16 years. Their main demographic and clinical characteristics are depicted in Table 1 that shows no significant differences between groups. Bicuspid aortic valve disease (36%) was the most common AR etiology followed by degenerative tricuspid aortic valve disease (31%). Nearly half of the patients (*n* = 43, 46%) were still asymptomatic, with a similar distribution in the two groups.

**Table 1:** Baseline characteristics of the study population

Characteristic	Total (*n* = 80)	Jet-related strain reduction (*n* = 34)	No regional strain reduction (*n* = 46)	*P*-value
Demographic				
Age (yr)	56 ± 16	59 ± 16	54 ± 16	0.790
Male sex	66 (83)	29 (85)	37 (80)	0.572
Body surface area (m^2^)	1.88 ± 0.18	1.87 ± 0.17	1.89 ± 0.20	0.708
GFR (mL/min/1.73 m^2^)	95 (78–108)	90 (80–108)	97 (78–108)	0.596
Cardiovascular risk factor^a^				
Hypertension	46 (63)	24 (73)	22 (55)	0.118
Dyslipidemia	26 (36)	15 (45)	11 (28)	0.111
Diabetes	5 (7)	4 (12)	1 (3)	0.169
Tobacco use	16 (22)	5 (15)	11 (28)	0.204
Medication^b^				
ACEi, ARB, ARNI	42 (65)	17 (63)	25 (66)	0.814
β-blocker	31 (48)	14 (52)	17 (45)	0.571
MRA	5 (8)	2 (7)	3 (8)	> 0.999
CCB	19 (29)	9 (33)	10 (26)	0.540
Diuretic	19 (29)	8 (30)	11 (29)	0.952
Symptomatic AR^c^	37 (54)	16 (55)	21 (53)	0.826
AR etiology				
Bicuspid	29 (36)	9 (27)	20 (44)	0.118
Degenerative	25 (31)	12 (35)	13 (28)	0.502
Annuloaortic dilation	14 (18)	7 (21)	7 (15)	0.532
Cusp prolapse	5 (6)	2 (6)	3 (7)	> 0.999
Rheumatic	3 (4)	1 (3)	2 (4)	> 0.999
Unknown	4 (5)	3 (9)	1 (2)	0.307
Eccentric AR	25 (31)	12 (35)	13 (28)	0.544

Values are presented as mean ± standard deviation, number (%), or median (interquartile range)

*GFR* glomerular filtration rate, *ACEi* angiotensin converting enzyme inhibitor, *ARB* angiotensin receptor blocker, *ARNI* angiotensin receptor-neprilysin inhibitor, *MRA* mineralocorticoid receptor antagonist, *CCB* calcium channel blocker, *AR* aortic regurgitation

^a^Seven missing values (one in the jet-related strain reduction group and six in the no regional strain reduction group). ^b^Fifteen missing values (seven in the jet-related strain reduction group and eight in the no regional strain reduction group). ^c^Eleven missing values (five in the jet-related strain reduction group and six in the no regional strain reduction group)

### Baseline echocardiographic assessment

Of all the patients included in the study, 43% were found to have jet-related LS reduction. In this group, the absolute LS values in the segments impacted by the regurgitant jet was a median of 10 percent points lower compared with nonaffected segments (corresponding to a 56% relative decrease). The most commonly affected segments were the basal septum (44%), mid inferolateral and/or anterolateral segment (24%), and basal inferior segment (12%).

Table 2 summarizes the echocardiographic data for both groups. All patients had moderate-to-severe or severe AR, and AR severity was similar between the two groups. The regurgitation was eccentric in a third of the cases but in the group without jet-related strain reduction, most of the eccentric jets (85%) were directed towards the anterior mitral leaflet. The vast majority of patients had preserved EF (with a mean LVEF of 55%) but reduced GLS (mean of –16%), with no significant differences between groups. LV diastolic function parameters did not differ between the groups either. In univariate and multivariate logistic regression, there were no significant predictors of the presence of jet-related strain reduction.

**Table 2:** Echocardiographic findings

Finding	Total (*n* = 80)	Jet-related strain reduction (*n* = 34)	No regional strain reduction (*n* = 46)	*P*-value
AR severity				
Moderate to severe	39 (49)	18 (53)	21 (46)	0.519
Severe	41 (51)	16 (47)	25 (54)	
EROA (mm^2^)	30 (22–38)	30 (21–40)	30 (23–38)	0.858
Regurgitant volume (mL)	62 (44–87)	63 (43–86)	60 (46–99)	0.726
Pressure half-time (msec)	335 (246–391)	350 (267–425)	309 (235–374)	0.317
LV dimension and systolic function				
LVESD (mm)	42 (38–49)	40 (36–47)	44 (38–51)	0.091
LVEDV (mL/m^2^)	98 (83–126)	94 (79–125)	99 (86–131)	0.365
LVEF (%)	55 ± 10	56 ± 8	54 ± 12	0.185
Reduced EF (< 50%)	25 (31)	7 (21)	18 (39)	0.077
GLS (%)	–16.0 ± 3.8	–16.5 ± 2.6	–15.5 ± 4.7	0.245
Reduced GLS (worse than –19%)	63 (79)	26 (76)	37 (80)	0.756
LV diastolic function				
E/e’	7 (6–9)	7 (6–8)	7 (5–9)	0.776
Left atrial volume (mL/m^2^)	38 (31–56)	44 (33–59)	38 (30–47)	0.279

Values are presented as number (%), median (interquartile range), or mean ± standard deviation

*AR* aortic regurgitation, *EROA* effective regurgitant orifice area, *LV* left ventricular, *LVESD* left ventricular end-systolic diameter, *LVEDV* left ventricular end-diastolic volume, *LVEF* left ventricular ejection fraction, *EF* ejection fraction, *GLS*, global longitudinal strain

### Postoperative echocardiographic assessment

From the total population, 36 patients (45%, 20 from the group with jet-related strain reduction and 16 from the group without it) underwent corrective surgery for AR. The most common procedure was aortic valve replacement performed in 30 patients (20 isolated valve replacements, six Bentall procedures, and four valve replacements with concomitant supracoronary aortic grafts); additionally, two patients had aortic valve repair and four underwent valve-sparing aortic root replacement (David procedure). Overall, 53% of patients had a mechanical valve implanted, with a similar distribution in both groups (50% in the group with jet-related strain reduction vs. 56% in the group without the regional pattern, *P* = 0.709).

In a postoperative assessment, a median of 6 months after surgery, LV end-diastolic volume had significantly reduced in the majority of cases (83%) but LVEF also worsened in around a third of cases (*n* = 13, 36%). Specifically, in the group that had jet-related strain reduction, when compared to the preoperative echocardiogram, the absolute difference in LS values between the impacted and nonimpacted LV segments decreased from a median of 10% to 4.7% in the preoperative versus postoperative TTE (*P* < 0.001).

Compared with patients who did not have the regional pattern of strain reduction, those who exhibited a jet-related strain reduction in the preoperative TTE were less likely to improve their GLS after surgery. They also had a tendency towards lower rates of recovery of EF after surgery (Table 3).

**Table 3:** Clinical characteristics and preoperative and postoperative echocardiographic findings of the subgroup of patients submitted to surgery

Variable	Jet-related strain reduction (*n* = 20)	No regional strain reduction (*n* = 16)	*P*-value
Clinical baseline characteristic			
Age	55 ± 18	54 ± 15	0.809
Male sex	19 (95)	14 (88)	0.574
AR symptoms^a^	11 (58)	7 (58)	0.981
Preoperative echocardiographic finding			
EROA (mm^2^)	34 (24–45)	30 (25–40)	0.947
Regurgitant volume (mL)	71 ± 29	91 ± 37	0.176
Moderate-to-severe AR	9 (45)	5 (31)	0.400
Severe AR	11 (55)	11 (69)	
LVESD (mm)	43 ± 8	52 ± 12	0.013^*^
LVEDV (mL/m^2^)	101 (89–122)	109 (92–151)	0.265
LVEF (%)	56 ± 7	51 ± 13	0.158
GLS (%)	–16.5 ± 2.8	–13.8 ± 5.3	0.088
Postoperative volume and systolic function			
LVEDV (mL/m^2^)	74 ± 20	70 ± 20	0.506
LVEDV reduction (≥ 15 mL/m^2^)	15 (75)	15 (94)	0.134
LVEF (%)	53 ± 10	54 ± 11	0.820
Improvement in LVEF (≥ 5)	3 (15)	7 (41)	0.056
Reduction in LVEF (≥ 5)	8 (40)	5 (31)	0.587
GLS (%)	–14.6 ± 4.9	–14.5 ± 2.8	0.945
Improvement in GLS (≥ 2.5)	2 (10)	6 (38)	0.049^*^

Values are presented as mean ± standard deviation, number (%), or median (interquartile range)

*AR* aortic regurgitation, *EROA* effective regurgitant orifice area, *LVESD* left ventricular end-systolic diameter, *LVEDV* left ventricular end-diastolic volume, *LVEF* left ventricular ejection fraction, *GLS* global longitudinal strain

^a^Five missing values (one in the jet-related strain reduction group and four in the no regional strain reduction group)

^*^P < 0.05

## Discussion

Myocardial strain represents the extent of myocardial deformation and is calculated as the percent change in myocardial length throughout the cardiac cycle [13]. While speckle tracking techniques have rendered it less angle-dependent, myocardial strain is still not an independent measure of myocardial contractility. It is influenced by hemodynamics, increasing with preload and decreasing with afterload as well as chamber geometry, decreasing with growing wall thickness. Finally, tissue characteristics, such as the presence of fibrosis or myocardial infiltration in infiltrative or storage diseases, have a significant impact on local myocardial deformation and often create regional strain patterns [14]. In the specific case of AR, it has been described that myocardial strain is initially increased as a consequence of volume overload. However, over time, the increasing wall stress ends up damaging the myocardium, leading to reduced contractility and lower strain [14, 15].

In this study of patients with moderate-to-severe or severe chronic AR, we demonstrated that often there is a regional strain pattern characterized by LS reduction in the myocardial segments that are directly hit by the impact of the regurgitant jet compared with the remaining segments. Furthermore, we found that this regional pattern became attenuated after corrective surgery for AR. These findings suggest that, aside from global loading conditions, myocardial deformation can also be affected by intracavitary flow dynamics.

Overall, in the subgroup of patients in which we were able to carry out a follow-up echocardiogram after corrective surgery for AR, we observed a reduction in LVEF in around a third of the patients, similar to the findings of Alashi et al. [16]. In this subgroup, we also found that the presence of a preoperative jet-related LS reduction was associated with less improvement in GLS after surgery.

We hypothesize that the myocardial segments undergoing the longstanding impact of the regurgitant jet might be subjected to a higher regional wall stress, eventually leading to reduced contractility and possibly even local fibrosis. In fact, regional LS reduction has been shown to be related with the presence of fibrosis in conditions such as myocardial infarction [17], hypertrophic cardiomyopathy [18, 19], and Fabry disease [20]. It has also been demonstrated that, in AR, the presence of myocardial fibrosis is associated with adverse remodeling and worse outcomes after valve surgery [21–23]. The development of focal fibrosis could therefore explain the regional strain impairment and its link with reduced recovery of systolic function after surgery observed in this study.

This is, to our knowledge, the first reported case series showing a direct negative effect of the aortic regurgitant jet’s impact on regional LV longitudinal function and possibly on postoperative LV remodeling as well. The strict exclusion criteria (which included any other significant valve disease, cardiomyopathy, coronary artery disease, and left bundle branch block) strongly reduce the possibility of confounding factors being at the origin of this regional strain pattern.

However, several limitations must be acknowledged. This was a retrospective study with a relatively small sample. In the surgical subgroup, coronary artery disease was excluded with invasive angiography as part of their preoperative assessment. However, in the nonsurgical subgroup, Computed tomography/invasive coronary angiograms were generally only performed in patients with a pretest probability of obstructive coronary artery disease of at least 5%, as per European Society of Cardiology recommendations [24]. Therefore, although the probability is quite low, we cannot completely exclude the presence of asymptomatic coronary artery disease in the nonsurgical subgroup. Additionally, several patients who underwent surgery had to be excluded from the postsurgery subgroup analysis because postoperative echocardiographic data were not available (usually because follow-up was continued in the patients’ local hospitals). Finally, the postoperative TTE was performed relatively early (6 months after surgery, on average), when abnormal septal motion related to pericardiotomy could still affect GLS.

## Conclusions

This study identifies an association between the parietal impact of the regurgitant jet in chronic AR and regional LS reduction. For patients submitted to corrective surgery, the presence of this regional pattern of LS impairment seems to be associated with worse postoperative LV remodeling.

Further studies with larger samples, longer clinical follow-up and particularly with cardiac magnetic resonance correlation may clarify whether underlying focal fibrosis might be at the origin of this regional pattern and assess the potential role of this regional pattern of LS impairment as a prognostic marker in chronic AR.

## Data Availability

Not applicable.

## Abbreviations

AR: Aortic regurgitation
D: Dimensional
EF: Ejection fraction
GLS: Global longitudinal strain
LS: Longitudinal strain
LV: Left ventricular
LVEF: Left ventricular ejection fraction
TTE: Transthoracic echocardiograms
